# Sliding inguinoscrotal hernia insinuating itself into the bladder, with calculi in the bladder and distal ureter

**DOI:** 10.1590/0100-3984.2015.0122

**Published:** 2017

**Authors:** Jose Domingos Contrera, Francisco Teixeira Cardoso Sobrinho

**Affiliations:** 1 IDI - Instituto de Diagnóstico por Imagem, Ribeirão Preto, SP, Brazil.; 2 Centro de Diagnóstico por Imagem, Parintins, AM, Brazil.

**Figure 1:**
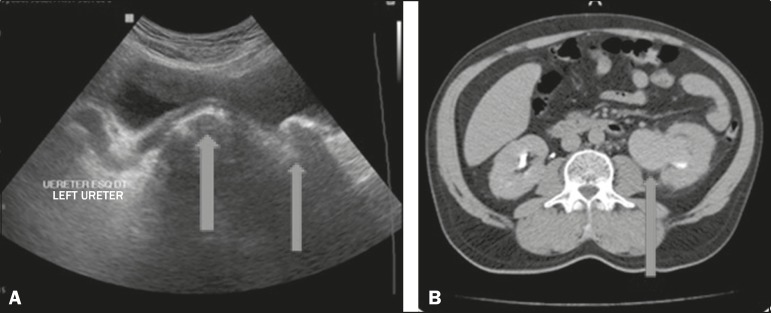
**A:** Ultrasound showing a calculus within the bladder and another calculus at the ureterovesical junction, with upstream dilation of the renal excretory pathway (arrows). **B:** Axial CT with delayed filling of the left renal pelvis (arrow).

**Figure 2:**
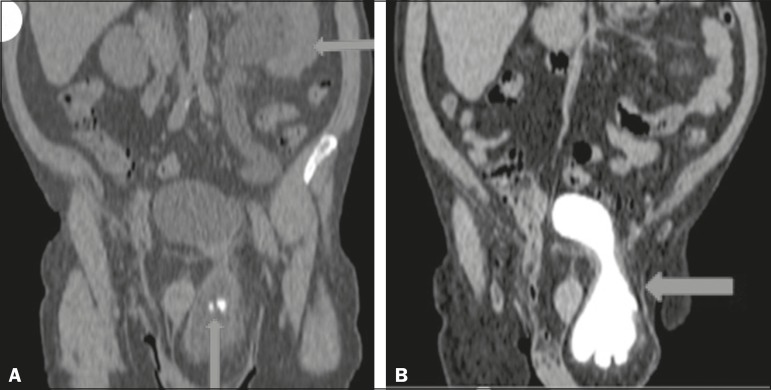
**A:** CT with coronal reconstruction showing a left-sided inguinoscrotal bladder hernia containing a calculus, another calculus being seen at the ureterovesical junction (seen on ultrasound), with upstream stasis, dilation, and tortuosity of the renal excretory pathway (arrows). **B:** Coronal CT reconstruction in the delayed phase confirming the left-sided inguinoscrotal hernia of the bladder (arrow).

Dear Editor,

A 55-year-old male presented with dysuria, polyuria, nocturia, and decreased urinary flow. The physical examination revealed a large left-sided inguinoscrotal hernia that was irreducible, together a slightly enlarged prostate. Laboratory tests showed mild anemia and pyuria, as well as elevated concentrations of urea and creatinine. Ultrasound showed a left-sided inguinoscrotal hernia that had insinuated itself into the bladder, with dilation of the distal ureteral segment and two calculi, one measuring approximately 2.2 cm, at the ureterovesical junction, and the other, of similar diameter, free within the bladder (Figure 1A). Computed tomography (CT) showed a filling delay and dilation of the renal pelvis (Figure 1B), as well as two calculi in the herniated bladder, one of which was at the left ureterovesical junction, with dilation of the renal excretory pathway in the coronal reconstruction (Figures 2A and 2B). On the basis of the information observed in the images, the decision was made to submit the patient to cystolithotomy in the region of the herniated bladder, removal of the calculi from the bladder and ureter (with Randall forceps), and correction of the inguinal hernia by the Lichtenstein technique. Subsequently, the patient evolved satisfactorily.

Bladder hernias, which are not uncommon, protrude into the femoral canal in women and into the inguinal canal in men^(1)^. In the past, inguinoscrotal hernias of the bladder were diagnosed intraoperatively or through excretory urography and produced complications such as urinary tract infection and obstructive uropathy^(2-4)^. The preoperative diagnosis of a hernia insinuating itself into the bladder is very important for the urologist, guiding the surgical planning^(5)^.

The examinations most commonly performed in order to diagnose inguinoscrotal hernias are ultrasound and CT^(6)^. Ultrasound is a noninvasive test, useful for identifying a herniated bladder and its components, as well as the continuity of the bladder with the non-herniated portion within the pelvis, and can be used in emergency settings. The use of multidetector CT facilitates the diagnosis and in traumatic cases can show other herniations in the abdominal wall, through coronal and sagittal reconstructions. Because of its high resolution, multidetector CT allows better visualization of the relationship between the bladder and the inferior epigastric vessels, thus facilitating the differentiation among direct inguinal, indirect inguinal, and femoral hernias^(7)^. Magnetic resonance imaging can be used in place of CT. The combination of ultrasound and magnetic resonance imaging is useful for the noninvasive, nonradiative evaluation of alterations in the scrotum^(8)^.

In the case presented here, we have described the importance of ultrasound for the diagnosis of a calculus within the lumen of the bladder and another at the ureterovesical junction, with upstream dilation of the renal excretory pathway, and for confirmation of the CT findings, which showed a calculus in the bladder and another at the left ureterovesical junction, contributing to the selection of appropriate practice and the success of the surgical procedure. In our review of the literature, we found no other reports of a ureteral calculus in a patient with inguinoscrotal hernia of the bladder.
